# Zeaxanthin pretreatment alleviates low-temperature stress-induced injury in flue-cured tobacco seedlings by modulating the xanthophyll cycle and photoprotection

**DOI:** 10.3389/fpls.2026.1922603

**Published:** 2026-09-04

**Authors:** Xi Mo, Weiguo Ye, Zhifeng Zeng, Guibin Luo, Shengxu Lin, Xinyan Lu, Yitong Li, Yuanyuan Wang, Huaiyuan Li, Shiyuan Deng

**Affiliations:** 1 College of Agriculture, South China Agricultural University, Guangzhou, China; 2 Center for Basic Experiments and Practical Training, South China Agricultural University, Guangzhou, China

**Keywords:** flue-cured tobacco, low-temperature stress, photoprotection, rewarming, xanthophyll cycle, zeaxanthin

## Abstract

Low temperature is a major abiotic stress that affects flue-cured tobacco (*Nicotiana tabacum* L.) seedlings during the early transplanting stage. Zeaxanthin (Zea), a xanthophyll pigment closely associated with the photosynthetic apparatus has been reported to alleviate chilling injury in crops. However, its physiological roles in flue-cured tobacco under alternating chilling and rewarming conditions remain unclear. In this study, seedlings of the flue-cured tobacco cultivar Yunyan 87 were pretreated with Zea at 0, 25, 50, 75, or 100 mg L^-1^ and subsequently exposed to low-temperature stress at 10 °C during both the day and night, followed by rewarming. Among the concentrations tested, 75 mg L^-1^ was the most effective in alleviating low-temperature injury. Compared with the low-temperature control (T1), Zea pretreatment increased plant height and maximum leaf area by 14.60%–34.03% and 21.90%–39.12%, respectively, during the stress period, and these beneficial effects persisted during rewarming. Zea pretreatment also alleviated membrane damage, as indicated by reduced malondialdehyde content and relative electrical conductivity, and improved photosynthetic performance. Across the sampling periods and Zea treatments, the maximum increases in chlorophyll content, net photosynthetic rate, and maximum photochemical efficiency of photosystem II (*Fv/Fm*) were 20.35%, 216.26%, and 70.78%, respectively, relative to T1. Targeted carotenoid analysis showed that exogenous Zea increased leaf zeaxanthin content by up to 624.61% and promoted xanthophyll-cycle de-epoxidation. It also increased non-photochemical quenching by 41.40% relative to T1 during the stress period, indicating an enhanced capacity for photoprotective thermal dissipation. Furthermore, Zea pretreatment strengthened antioxidant and osmotic adjustment, as evidenced by increased catalase, peroxidase, and superoxide dismutase activities and higher soluble protein and proline contents. Hormone analysis further revealed increases in indole-3-acetic acid, jasmonic acid, and brassinosteroid contents, accompanied by a reduction in abscisic acid content. Collectively, these findings indicate that Zea pretreatment enhances the low-temperature tolerance of flue-cured tobacco seedlings and that this enhancement is associated with xanthophyll cycle modulation, improved photosynthetic performance, and strengthened antioxidant, osmotic, and hormonal adjustments.

## 1 Introduction

The chloroplast photosynthetic apparatus is among the earliest and most direct sites at which plants perceive and respond to low-temperature stres**s** (Wang et al., 2024). In temperature-sensitive crops such as rice (Xiong et al., 2025), maize (Feng et al., 2023), and tobacco (Lin et al., 2025), chilling stress disrupts chlorophyll biosynthesis, destabilizes the PSII reaction center, reduces the maximum photochemical efficiency of PSII (*Fv/Fm*), and inhibits key carbon-assimilation enzymes such as Rubisco. Consequently, maintaining a balance between light-energy capture and carbon assimilation is a major challenge for plants under low-temperature conditions (Thomashow, 1999). When the amount of absorbed light energy exceeds the capacity for its downstream utilization, energy capture becomes uncoupled from energy consumption, resulting in cellular energy imbalance. This imbalance can impair antioxidant defenses, including the activity of superoxide dismutase (SOD), and promote the excessive accumulation of reactive oxygen species (ROS) (Sui, 2015; Wang et al., 2022; Hu et al., 2025). Excessive ROS accumulation further accelerates lipid peroxidation, increases membrane permeability, and induces electrolyte leakage (Zhou et al., 2022). Therefore, maintaining photosynthetic integrity and redox homeostasis during the early stages of chilling stress is essential for improving plant cold tolerance.

Several exogenous compounds, including glutamine, catechin, and magnesium, have been shown to alleviate chilling injury in flue-cured tobacco (Li et al., 2023; Lin et al., 2025; Zhu et al., 2025). These compounds generally function by enhancing basal metabolic processes or supplying essential nutrients. However, effective strategies for protecting the core components of the photosynthetic membrane system remain insufficiently understood. In particular, the mechanisms that coordinate energy dissipation and free-radical scavenging under chilling stress have not been fully elucidated. Zeaxanthin is a naturally occurring carotenoid whose conjugated double-bond system enables it to directly scavenge free radicals and protect lipids and DNA from oxidative damage (Sanlier et al., 2024). More importantly, zeaxanthin is a key component of plant non-photochemical quenching (*NPQ*) and accumulates rapidly in response to excess excitation energy within the photosynthetic apparatus (Demmig-Adams et al., 2013, 2020, 2025; Murchie and Ruban, 2020). These properties make zeaxanthin a promising candidate for agricultural applications. Nevertheless, the mechanisms by which exogenous zeaxanthin enhances the photoprotective capacity of flue-cured tobacco seedlings remain unclear.

In other crops, zeaxanthin has been reported to coordinate multiple stress-response pathways. Under low temperature stress, it activates *NPQ* by upregulating the *CaZEP* gene in pepper (Pu et al., 2024). Under drought stress, it promotes ABA accumulation and enhances antioxidant enzyme activity in apple (Tian, 2024). Under high temperature stress, it helps maintain the net photosynthetic rate in chickpea (Kumar et al., 2020). However, the effects of exogenous zeaxanthin on the metabolic and physiological responses of flue-cured tobacco to chilling stress remain unclear. It is also unknown whether zeaxanthin can maintain coordination between the photosynthetic and antioxidant systems during the recovery phase following rewarming.

To address these questions, flue-cured tobacco seedlings were used as the experimental material in this study. We conducted a controlled experiment to quantify the short-term physiological responses of the seedlings treated with different concentrations of exogenous zeaxanthin during chilling stress and subsequent rewarming. We focused on chlorophyll fluorescence parameters, antioxidant enzyme activities, and targeted carotenoid metabolite profiles. Structural equation modeling (SEM) was subsequently used as an exploratory approach to integrate these physiological modules. To the best of our knowledge, this is the first study to evaluate the effects of exogenous zeaxanthin on flue-cured tobacco under an integrated chilling-rewarming regime and to link targeted xanthophyll-cycle metabolite profiling with whole-plant physiological performance.

Accordingly, we proposed three hypotheses: (1) exogenous zeaxanthin rapidly increases endogenous zeaxanthin content in flue-cured tobacco and enhances xanthophyll-cycle-dependent photoprotection, thereby maintaining PSII stability; (2) under chilling stress, zeaxanthin coordinately strengthens photoprotective energy dissipation and antioxidant enzyme activity, thereby alleviating membrane damage and reducing excessive malondialdehyde (MDA) accumulation; and (3) an optimal concentration of exogenous zeaxanthin alleviates oxidative damage during chilling stress and accelerates the recovery of photosynthetic carbon assimilation after rewarming, potentially through the regulation of specific metabolic pathways.

## 2 Materials and methods

### 2.1 Plant materials

This experiment was conducted from March to June 2025 in the Plant Physiology Instrument Room of the Center for Basic Experiments and Practical Training at South China Agricultural University. The flue-cured tobacco cultivar Yunyan 87 was used in this study. Seedlings were grown under natural conditions, and healthy plants with uniform growth were selected for transplanting at the five- to six-true-leaf stage. Each seedling was transplanted into a pot containing 5 kg of soil. Each pot was 17 cm in height and 16 cm in diameter, and one seedling was planted per pot.

Because zeaxanthin is lipophilic and poorly soluble in water, the working solutions were prepared as follows. Zeaxanthin was first dissolved in a small volume of absolute ethanol and then diluted with distilled water containing 0.01% (v/v) Tween-80 (catalogue no. 1716ML500; BioFroxx, neoFroxx GmbH, Germany) to prepare a 100 mg L^−1^ stock solution. The stock solution was subsequently diluted with the same Tween-80 solution to obtain the final treatment concentrations of 0, 25, 50, 75, and 100 mg L^−1^. The final ethanol concentration was adjusted to 1% (v/v) in all working solutions. CK and T1 plants were sprayed with an equal volume of the same solvent solution without zeaxanthin.

The soil used in the experiment was substrate soil with an m (N): m (P_2_O_5_): m (K_2_O) ratio of 16:8:16 and a pH of 6.7. The compound fertilizer used in the experiment had an m (N): m (P_2_O_5_): m (K_2_O) ratio of 12:8:16 and was manufactured by Shanxi Qinchuan Fertilizer Industry Co., Ltd. It was applied at a rate of 10 g per pot. Zeaxanthin (analytical reagent grade, purity ≥ 85%; molecular formula, C_40_H_56_O_2_) was purchased from Macklin Biochemical Co., Ltd. (Shanghai, China).

### 2.2 Experimental design

The experiment consisted of six treatments. CK served as the control treatment, in which plants were sprayed with distilled water and maintained under normal-temperature conditions. The other five treatments consisted of zeaxanthin applications under low-temperature stress: T1 (0 mg L^-1^), T2 (25 mg L^-1^), T3 (50 mg L^-1^), T4 (75 mg L^-1^), and T5 (100 mg L^-1^). Each treatment included three replicates, with 10 pots per replicate, resulting in a total of 180 pots.

Ten days after transplanting, zeaxanthin was applied as a foliar spray at a rate of 2 mL per plant between 18:00 and 18:30 h. The leaves were sprayed until droplets formed on both the adaxial and abaxial surfaces, without runoff. Foliar spraying was performed for three consecutive days. After spraying, the seedlings were transferred to a programmable artificial climate chamber (TRP-1000D, Changzhou Dedu Precision Instrument Co., Ltd.) and subjected to low-temperature stress at 10 °C during both the day and night. After 9 days of low-temperature treatment, the plants were transferred to recovery conditions at 28 °C/20 °C day/night temperatures. Control plants were maintained at 28 °C/20 °C and sprayed with the same volume of water.

The pots were arranged in a completely randomized design within the chamber and repositioned every three days to minimize microenvironmental effects. Relative humidity was maintained at 80% ± 5%. All other environmental conditions were kept constant among treatments, including a photosynthetic photon flux density (PPFD) of approximately 220 μmol m^-2^ s^-1^ and a photoperiod of 14 h light/10 h dark.

### 2.3 Measurement parameters and methods

For each treatment, three tobacco plants with uniform growth were selected for sampling and measurement. At each sampling time point, the fifth fully expanded leaf from the shoot apex was collected. Measurements were performed at 0, 3, 6, and 9 days of low-temperature stress and at 3 and 6 days after the onset of recovery.

#### 2.3.1 Agronomic traits

Plant height, leaf length (L), and leaf width (W) were measured at each sampling time point. Leaf area (LA) was calculated using the following equation: LA = L (cm) × W (cm) × 0.6345.

#### 2.3.2 Photosynthetic parameters and chlorophyll fluorescence parameters

Photosynthetic and chlorophyll fluorescence parameters were measured on clear days between 09:00 and 11:00 h using a CIRAS-II portable photosynthetic system (PP-Systems, UK) and an FMS2 pulse-modulated fluorometer (Hansatech Instruments Ltd., King’s Lynn, Norfolk, UK), respectively. The net photosynthetic rate (*Pn*), stomatal conductance (*Gs*), and intercellular CO_2_ concentration (*Ci*) were measured using the CIRAS-II system under a PPFD of 1000 μmol m^−2^ s^−1^.

Before chlorophyll fluorescence measurements, the leaves were dark-adapted for 30 min using dark-adaptation clips. The maximum photochemical efficiency of photosystem II (PSII), expressed as *Fv/Fm*, was then determined. Following the *Fv/Fm* measurement, the same leaves were illuminated with actinic light at 1000 μmol m^−2^ s^−1^ for 10 min to reach a steady state. Saturating pulses of 0.8 s were subsequently applied to determine the maximum chlorophyll fluorescence of the light-adapted leaves (*Fm′*). Non-photochemical quenching (*NPQ*) was calculated as follows: *NPQ* = (*Fm* –*Fm′*)/*Fm′* (Maxwell and Johnson, 2000).

#### 2.3.3 Chlorophyll content

Leaf discs were collected using a punch sampler. Chlorophyll content (Chl) was determined using the spectrophotometric method described by Lichtenthaler and Wellburn (1983).

#### 2.3.4 Antioxidant enzyme activities, stress-related physiological indices, and endogenous hormones

Superoxide dismutase (SOD) activity was determined using the nitroblue tetrazolium (NBT) photochemical reduction method (Shen et al., 1996). Peroxidase (POD) activity was determined using the guaiacol method, and catalase (CAT) activity was determined using the ultraviolet absorption method (Li, 2000). Leaf relative electrolyte conductivity (REC) was measured according to the method described by Lutts et al. (1996).

Total antioxidant capacity (TAOC), malondialdehyde (MDA), proline (Pro), and soluble protein (SP) contents were determined using commercial assay kits purchased from Suzhou Grace Biotechnology Co., Ltd. (Suzhou, China). The contents of abscisic acid (ABA), brassinosteroid (BR), indole-3-acetic acid (IAA), and jasmonic acid (JA) were determined using commercial enzyme-linked immunosorbent assay (ELISA) kits (catalogue nos. XYPA-10515 for ABA, XYPA-10814 for BR, XYPA-10875 for IAA, and XYPA-10815 for JA; Shanghai Xuanya Biotechnology Co., Ltd., Shanghai, China). All four kits were based on the sandwich ELISA principle. Briefly, leaf samples were homogenized in phosphate-buffered saline (PBS), and the supernatants were analyzed according to the manufacturer’s instructions. Absorbance was measured at 450 nm, and hormone concentrations were calculated from standard curves. The coefficient of determination (R^2^) was ≥ 0.99 for all four standard curves. Both the intra-assay and inter-assay coefficients of variation were below 10%.

#### 2.3.5 Targeted metabolomics of carotenoids

For targeted carotenoid metabolomic analysis, leaf samples were ground into a powder using a ball mill at 30 Hz for 1 min. A 50 mg aliquot of each ground sample was accurately weighed using an analytical balance. The sample was extracted with 0.5 mL of an n-hexane/acetone/ethanol mixture (1:1:1, v/v/v) containing 0.01% butylated hydroxytoluene (BHT). The mixture was vortexed for 20 min at room temperature and then centrifuged at 12,000 rpm for 5 min at 4 °C. The supernatant was collected, and the extraction was repeated once. The supernatants from the two extractions were combined concentrated, and reconstituted in 150 μL of dichloromethane. After filtration through a 0.22 μm membrane, the extract was transferred to amber sample vials for LC-MS/MS analysis.

Data were acquired using an ultra-performance liquid chromatography system (UPLC; ExionLC™ AD) coupled to a tandem mass spectrometer. Chromatographic separation was performed on a YMC C30 column (3 μm, 100 mm × 2.0 mm i.d.). Mobile phase A consisted of methanol/acetonitrile (1:3, v/v) containing 0.01% BHT and 0.1% formic acid. Mobile phase B consisted of methyl tert-butyl ether containing 0.01% BHT. The elution gradient was as follows: 0 min, A/B = 100:0 (v/v); 3 min, 100:0; 5 min, 30:70; 9 min, 5:95; 10 min, 100:0; and 11 min, 100:0. The flow rate was 0.8 mL min^-1^, the column temperature was maintained at 28 °C, and the injection volume was 2 μL.

Mass spectrometric analysis was performed using an atmospheric pressure chemical ionization (APCI) source. The APCI source temperature was set to 350 °C, and the curtain gas pressure was maintained at 25 psi. In the Q-Trap 6500+ system, each ion transition was monitored using optimized declustering potential (DP) and collision energy (CE) parameters.

Quality control procedures were implemented throughout the LC–MS/MS analysis. A pooled quality-control (QC) sample, prepared by mixing equal aliquots of all samples, was injected in triplicate to monitor the stability of the analytical system. An isotope-labeled internal standard ([13C10]-β-carotene, 2 ppm) was used for signal monitoring but was not used for quantification. Method validation showed good linearity within the respective calibration ranges for all target carotenoids. The intra-day relative standard deviations (RSDs) ranged from 0.08% to 3.85%, the inter-day RSDs ranged from 0.72% to 19.07%, and the spike recoveries ranged from 61.4% to 116.5%. Among the 21 carotenoids detected in the pooled QC samples, the RSDs of the peak areas ranged from 0.35% to 25.60%, with a median value of 5.15%. In total, 90.5% of the analytes had RSDs of ≤ 20%, indicating that the analytical system was stable and that the metabolomic data were reliable.

### 2.4 Statistical analyses

Raw data were organized and processed using Microsoft Excel 2024 (Microsoft Corp., Redmond, WA, USA). Statistical analyses were performed using SPSS 22.0 (IBM Corp., Armonk, NY, USA). Three biological replicates were analyzed for each treatment at each sampling time point, and each replicate consisted of leaf tissue pooled from three uniform plants (n = 3).

Before analysis of variance, data normality and homogeneity of variance were assessed using the Shapiro–Wilk test and Levene’s test, respectively. Differences among treatments at each sampling time point were assessed using one-way analysis of variance (ANOVA), followed by Duncan’s multiple-range test at *p* < 0.05. Because plants were sampled destructively at each time point and different plants were measured at different time points, two-way ANOVA was additionally performed, with treatment and sampling time as fixed factors, to evaluate their main effects and interaction.

Significant effects of treatment, sampling time, and the treatment × sampling time interaction were detected for the key traits (all *p* < 0.001). Detailed statistical results are presented with the corresponding findings in Sections 3.1–3.3. Correlation analyses were performed using the Metware Cloud bioinformatics platform. Structural equation modeling (SEM) was conducted using SPSSAU (QingSi Technology Co., Ltd., Beijing, China) based on 108 observations, corresponding to six treatments × six sampling times × three biological replicates.

Five latent variables, namely photosynthetic performance, membrane lipid peroxidation and injury, antioxidant defense, hormonal balance, and osmotic regulation, were constructed from the corresponding observed physiological and agronomic indicators identified through correlation analysis. Model parameters were estimated using maximum likelihood. Model fit was evaluated based on χ2/df, the comparative fit index (CFI), the normed fit index (NFI), and the root mean square error of approximation (RMSEA), using the following acceptance criteria: χ2/df < 3, CFI > 0.90, NFI > 0.90, and RMSEA < 0.10. SEM was used as an exploratory tool to summarize the covariance structure among physiological modules and was not interpreted as evidence of causal relationships. All other figures were generated using Origin 2024b (OriginLab Corp., Northampton, MA, USA).

## 3 Results

### 3.1 Effects of zeaxanthin pretreatment on the growth of flue-cured tobacco seedlings under low-temperature stress and during recovery

**Figure 1:**
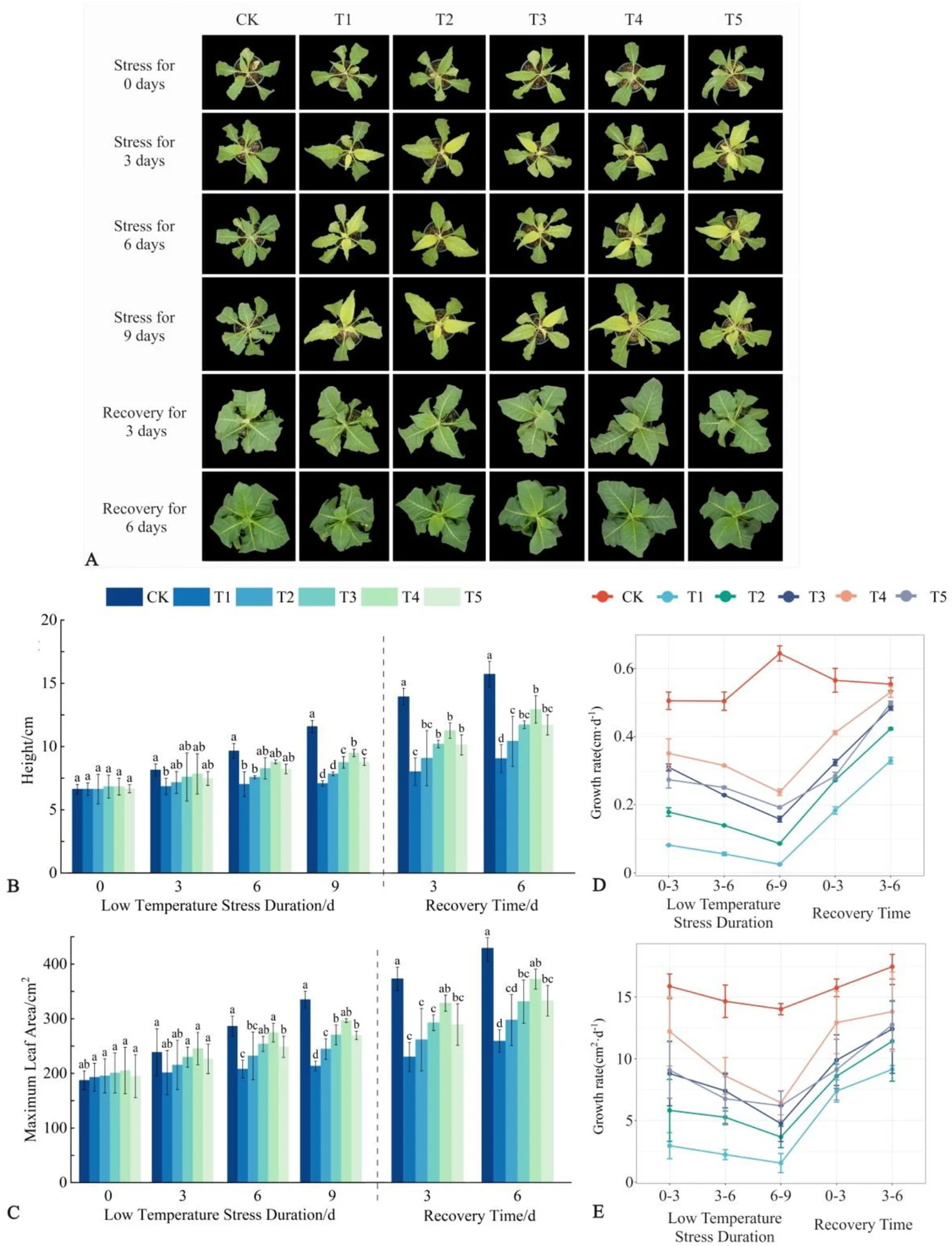
Effects of zeaxanthin pretreatment on the growth of flue-cured tobacco seedlings under low-temperature stress and during the recovery period. **(A)** Representative phenotypes of flue-cured tobacco seedlings. **(B)** Plant height. **(C)** Maximum leaf area. **(D)** Plant height growth rate. **(E)** Maximum leaf area growth rate. Error bars represent the standard error (SE). Different lowercase letters indicate statistically significant differences among treatments at the same growth stage (*p* < 0.05; ANOVA followed by Duncan’s multiple range test).

As shown in **Figure 1**, no apparent differences in plant morphology or agronomic traits were observed among treatments before the onset of low-temperature stress. Low-temperature exposure markedly inhibited seedling growth, with the greatest inhibition observed in T1. Zeaxanthin pretreatment significantly alleviated this growth inhibition. Plant height, maximum leaf area, and their corresponding growth rates generally increased and then decreased with increasing zeaxanthin concentration, with T4 showing the strongest effect throughout the sampling period. Compared with T1, T4 increased plant height and maximum leaf area by 14.60%–34.03% and 21.90%–39.12%, respectively, during the stress period.

During recovery, T1 seedlings exhibited only partial regreening and remained smaller and less vigorous than CK seedlings. Zeaxanthin pretreatment significantly promoted growth recovery, particularly in T4. Although plant height and maximum leaf area in T4 remained lower than those in CK, they were significantly higher than those in T1, increasing by 40.53%–42.89% and 42.70%–43.64%, respectively. Two-way ANOVA revealed significant main effects of treatment and sampling time, as well as a significant treatment × sampling time interaction, on both plant height and maximum leaf area. For plant height, the *F* values for treatment, sampling time, and their interaction were 147.900, 71.618, and 6.419, respectively. For maximum leaf area, the corresponding *F* values were 155.447, 55.044, and 4.763, respectively. All effects were significant at *p* < 0.001, indicating that the effects of zeaxanthin pretreatment varied significantly across sampling times.

### 3.2 Effects of zeaxanthin pretreatment on photosynthetic traits of flue-cured tobacco seedlings under low-temperature stress and during recovery

As shown in **Table 1**, low-temperature stress significantly reduced Chl, *Pn*, *Gs*, and *Fv/Fm*, while increasing *Ci*. The most pronounced changes were generally observed in T1. Compared with T1, zeaxanthin pretreatment significantly improved photosynthetic performance and reduced *Ci*. T4 generally produced the strongest response, increasing Chl, *Pn*, and *Gs* by 20.35%, 216.26%, and 418.37%, respectively. The greatest increase in *Fv/Fm* was observed in T3, where it was 70.78% higher than that in T1. *Ci* showed the greatest reduction in T4 and was 29.34% lower than that in T1.

**Table 1:** Effects of zeaxanthin pretreatment on photosynthetic traits of flue-cured tobacco seedlings under low-temperature stress and during recovery.

Sampling time	Treatment	Chl (mg g^-1^)	*Pn* (μmol m^-2^ s^-1^)	*Gs* (mol m^-2^ s^-1^)	*Ci* (μmol mol^-1^)	*Fv*/*Fm*	*NPQ*
Low-temperature stress for 6 days	CK	1.213 ± 0.057a	12.209 ± 0.641a	0.491 ± 0.021a	279.173 ± 8.426e	0.766 ± 0.016a	0.487 ± 0.015e
	T1	0.737 ± 0.027c	2.158 ± 0.086f	0.049 ± 0.008e	404.501 ± 12.758a	0.397 ± 0.017c	1.483 ± 0.032d
	T2	0.871 ± 0.038b	3.555 ± 0.057e	0.091 ± 0.012d	378.146 ± 9.211b	0.636 ± 0.070b	1.817 ± 0.025c
	T3	0.873 ± 0.027b	4.847 ± 0.038f	0.159 ± 0.006c	341.765 ± 1.095c	0.678 ± 0.047b	1.900 ± 0.026b
	T4	0.887 ± 0.020b	6.825 ± 0.161b	0.254 ± 0.003b	312.743 ± 2.412d	0.649 ± 0.007b	2.097 ± 0.025a
	T5	0.846 ± 0.011b	5.456 ± 0.190c	0.232 ± 0.004b	328.149 ± 5.496cd	0.646 ± 0.024b	2.047 ± 0.040a
Rewarming for 3 days	CK	1.302 ± 0.008b	12.853 ± 1.523a	0.468 ± 0.040a	238.154 ± 3.022d	0.779 ± 0.013a	0.497 ± 0.072d
	T1	1.094 ± 0.008d	4.598 ± 0.630d	0.105 ± 0.004d	373.907 ± 16.780a	0.626 ± 0.051c	1.527 ± 0.040c
	T2	1.296 ± 0.008b	6.658 ± 0.436c	0.147 ± 0.024d	325.903 ± 1.884b	0.645 ± 0.002c	1.620 ± 0.020b
	T3	1.290 ± 0.007b	7.959 ± 0.185bc	0.275 ± 0.012c	322.422 ± 11.513b	0.650 ± 0.033c	1.683 ± 0.015b
	T4	1.386 ± 0.004a	9.397 ± 0.289b	0.347 ± 0.015b	261.485 ± 8.223cd	0.749 ± 0.02ab	1.797 ± 0.025a
	T5	1.270 ± 0.014c	9.031 ± 1.130b	0.339 ± 0.011bc	273.742 ± 6.888c	0.716 ± 0.023b	1.757 ± 0.035a

Values are means ± SE (n = 3) for six treatments at two sampling times. Different letters indicate significant differences among treatments at the same growth stage (*p* < 0.05; one-way ANOVA followed by Duncan’s multiple range test).

Rewarming partially restored photosynthetic performance in all treatments. However, Chl, *Pn*, *Gs*, and *Fv/Fm* in T1 remained significantly lower than those in CK. Zeaxanthin pretreatment further improved these parameters during recovery, with T4 again showing the strongest effect. In T4, Chl, *Pn*, *Gs*, and *Fv/Fm* were 26.69%, 230.48%, 104.37%, and 19.65% higher than those in T1, respectively. The significant treatment × sampling time interactions detected for *Pn* and *Fv/Fm* indicated that the effects of zeaxanthin pretreatment differed between the stress and recovery periods.

*NPQ* increased significantly under low-temperature stress and was further enhanced by zeaxanthin pretreatment. T4 showed the highest *NPQ*, which was 41.40% higher than that in T1 during stress and remained 17.70% higher during recovery. Two-way ANOVA revealed significant effects of treatment, sampling time, and their interaction on *Pn*, *Fv/Fm*, and *NPQ*. For *Pn*, the *F* values for treatment, sampling time, and their interaction were 408.809, 423.082, and 22.785, respectively. The corresponding values were 183.090, 165.666, and 18.811 for *Fv/Fm* and 1310.711, 900.198, and 61.039 for *NPQ*. All effects were significant at *p* < 0.001, indicating that zeaxanthin treatment and sampling time jointly influenced photosynthetic responses.

### 3.3 Effects of zeaxanthin pretreatment on the antioxidant system of flue-cured tobacco seedlings under low-temperature stress and during recovery

**Figure 2:**
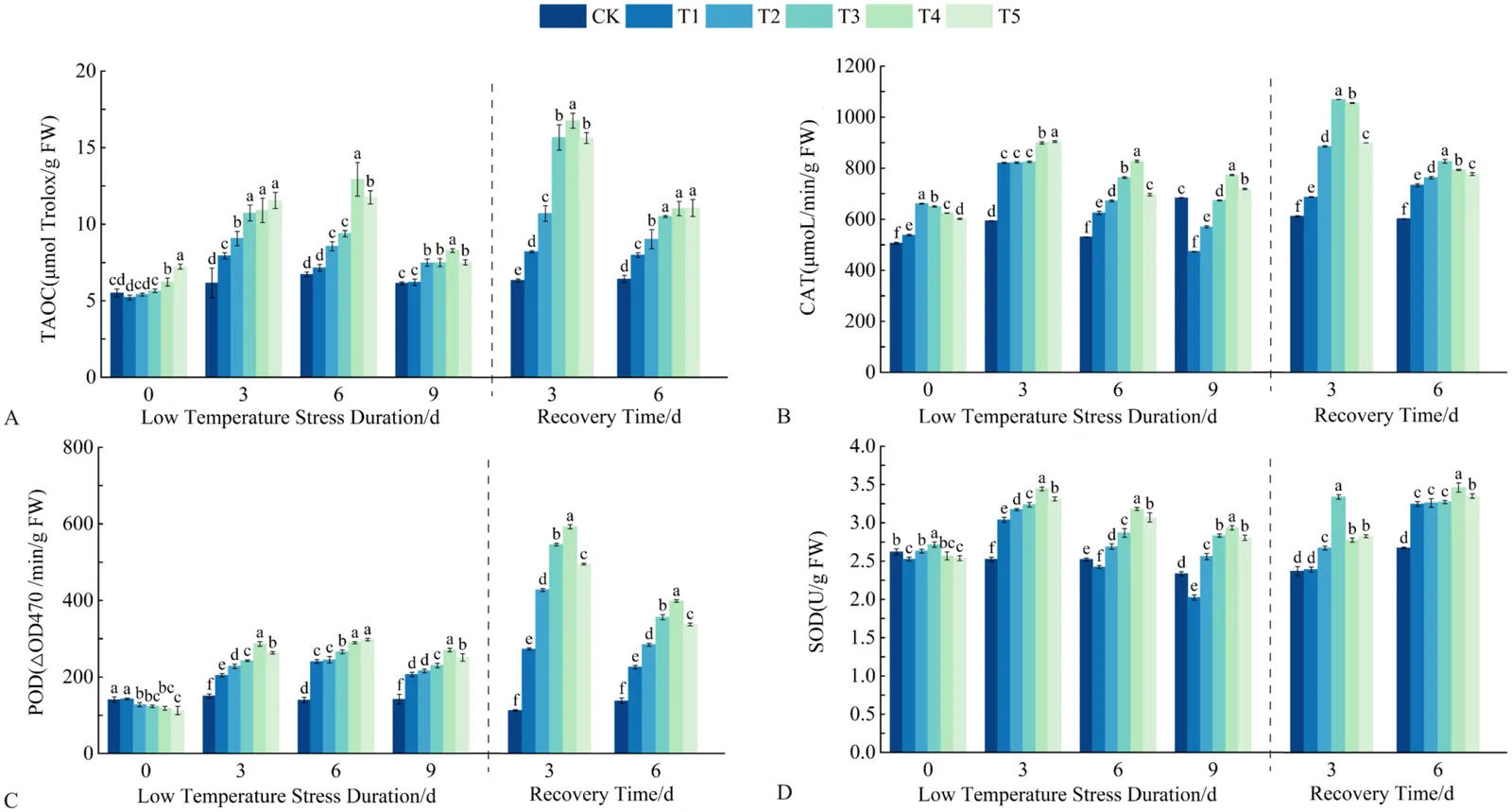
Effects of zeaxanthin pretreatment on the antioxidant system of flue-cured tobacco seedlings under low-temperature stress and during the recovery period. **(A)** Total antioxidant capacity (TAOC). **(B)** Catalase (CAT) activity. **(C)** Peroxidase (POD) activity. **(D)** Superoxide dismutase (SOD) activity. Error bars represent the standard error (SE). Different lowercase letters indicate statistically significant differences among treatments at the same growth stage (*p* < 0.05; ANOVA followed by Duncan’s multiple range test).

As shown in **Figure 2**, after 3 days of low-temperature stress, TAOC and the activities of CAT, POD, and SOD were significantly higher in T1 than in CK, indicating activation of the antioxidant defense system. CAT and SOD activities generally declined as the stress period progressed. Compared with T1, zeaxanthin pretreatment significantly enhanced antioxidant capacity. The responses generally exhibited a single-peak pattern with increasing zeaxanthin concentration, and T4 produced the strongest overall effect during the stress period. Compared with T1, T4 increased TAOC, CAT, POD, and SOD by 33.28%–80.76%, 9.53%–63.41%, 20.41%–40.02%, and 13.37%–45.10%, respectively.

During recovery, all four antioxidant parameters remained higher in T1 than in CK. Zeaxanthin pretreatment further increased these parameters, although the treatment producing the strongest response varied among antioxidant enzymes and recovery time points. T4 produced the greatest increases in TAOC and POD on day 3 of recovery and in POD and SOD on day 6 of recovery, whereas T3 produced the highest CAT activity on both recovery time points. These results indicate that zeaxanthin enhanced antioxidant defense during both low-temperature stress and recovery, although treatment effects varied among antioxidant components and sampling times.

### 3.4 Effects of zeaxanthin pretreatment on membrane lipid peroxidation and osmoregulatory substances in flue-cured tobacco seedlings under low-temperature stress and during recovery

**Figure 3:**
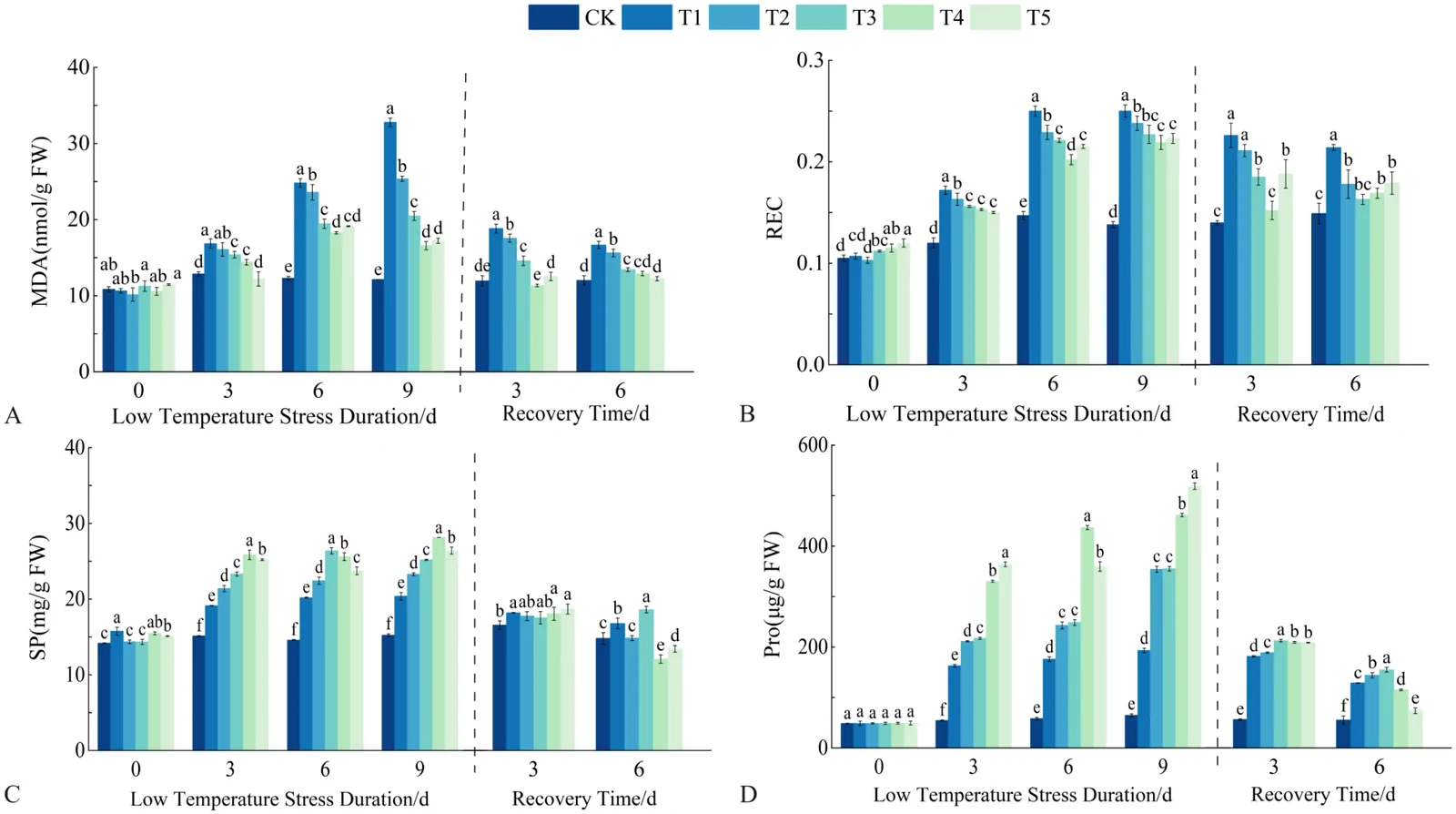
Effects of zeaxanthin pretreatment on membrane lipid peroxidation and osmotic adjustment substances in flue-cured tobacco seedlings under low-temperature stress and during the recovery period. **(A)** Malondialdehyde (MDA) content. **(B)** Relative electrolyte conductivity (REC). **(C)** Soluble protein (SP) content. **(D)** Proline (Pro) content. Error bars represent the standard error (SE). Different lowercase letters indicate statistically significant differences among treatments at the same growth stage (*p* < 0.05; ANOVA followed by Duncan’s multiple range test).

As shown in **Figure 3**, low-temperature stress significantly increased MDA content and REC, and both parameters were markedly higher in T1 than in CK. Zeaxanthin pretreatment significantly reduced membrane lipid peroxidation and membrane injury relative to T1. T4 showed the strongest protective effect, particularly during the later stages of low-temperature stress. Compared with T1, MDA content in T4 was 26.48% and 48.49% lower on days 6 and 9 of stress, respectively, whereas REC was 32.74% and 12.71% lower, respectively.

During recovery, MDA content and REC declined in all treatments but remained significantly higher in T1 than in CK. By day 3 of recovery, both parameters in T4 were significantly lower than those in T1 and did not differ significantly from those in CK. Two-way ANOVA confirmed significant main effects of treatment and sampling time, together with a significant treatment × sampling time interaction, on MDA content. The *F* values for treatment, sampling time, and their interaction were 920.956, 565.517, and 83.909, respectively, and all effects were significant at *p* < 0.001. This interaction indicated that the protective effect of zeaxanthin against membrane lipid peroxidation changed significantly over time.

Low-temperature stress also significantly increased SP and Pro contents. Zeaxanthin pretreatment further enhanced the accumulation of both osmolytes, although the treatment producing the strongest response differed between the two substances and among sampling times. T4 produced the largest increase in SP content, which was 26.85%–38.00% higher than that in T1. The greatest increases in Pro content occurred in T5 on days 3 and 9 of low-temperature stress, reaching 123.45% and 148.48% above the T1 level, respectively. During recovery, SP and Pro contents declined but remained higher than those in CK, with T3 showing the strongest Pro response. These results indicate that zeaxanthin promoted osmotic adjustment during low-temperature stress and maintained osmotic protection during recovery.

### 3.5 Effects of zeaxanthin pretreatment on endogenous hormone contents in flue-cured tobacco leaves under low-temperature stress and during recovery

**Figure 4:**
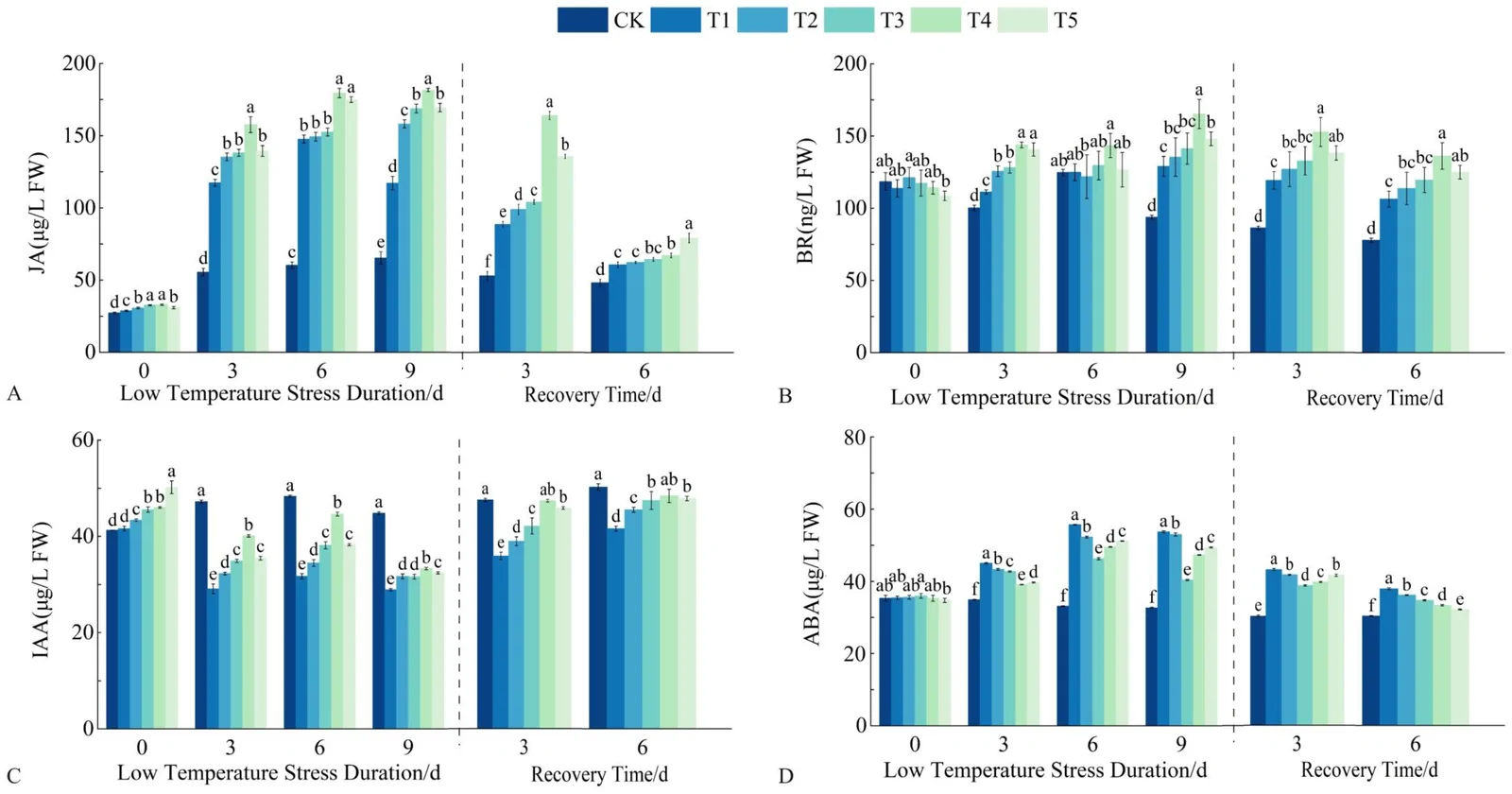
Effects of zeaxanthin pretreatment on endogenous hormone levels in flue-cured tobacco leaves under low-temperature stress and during the recovery period. **(A)** Jasmonic acid (JA) content. **(B)** Brassinosteroid (BR) content. **(C)** Indole-3-acetic acid (IAA) content. **(D)** Abscisic acid (ABA) content. Error bars represent the standard error (SE). Different lowercase letters indicate statistically significant differences among treatments at the same growth stage (*p* < 0.05; ANOVA followed by Duncan’s multiple range test).

As shown in **Figure 4**, low-temperature stress significantly increased JA, BR, and ABA contents but reduced IAA content relative to CK. Zeaxanthin pretreatment significantly modified these responses by increasing JA, BR, and IAA contents and generally reducing ABA content. JA, BR, and IAA generally exhibited single-peak responses to increasing zeaxanthin concentration, with the strongest responses generally observed in T4. Compared with T1, the contents of JA, BR, and IAA in T4 increased by 21.59%–55.03%, 14.84%–29.30%, and 15.24%–40.73%, respectively. In contrast, ABA exhibited an opposite concentration-dependent response, with the lowest levels primarily observed in T3.

During recovery, JA, BR, and ABA contents generally decreased, whereas IAA content increased in all treatments. JA and BR contents remained significantly higher in zeaxanthin-pretreated plants than in T1. In T4, JA content was 85.00% higher than that in T1 on day 3 of recovery, whereas BR content was approximately 28% higher on days 3 and 6 of recovery. IAA content in T4 recovered to a level comparable to that in CK and was significantly higher than that in T1.

In addition, ABA content was negatively correlated with Gs across treatments. This correlation was significant on days 3 (r = −0.838, *p* < 0.01) and 6 (r = −0.871, *p* < 0.01) of low-temperature stress and on days 3 (r = −0.832, *p* < 0.01) and 6 (r = −0.586, *p* < 0.05) of recovery. However, the correlation was not significant on day 0 (r = −0.110, *p* = 0.663) or day 9 (r = −0.381, *p* = 0.119) of low-temperature stress (n = 18 at each sampling time point).

### 3.6 Correlation analysis of physiological traits and structural equation model-based pathway analysis of zeaxanthin action

**Figure 5:**
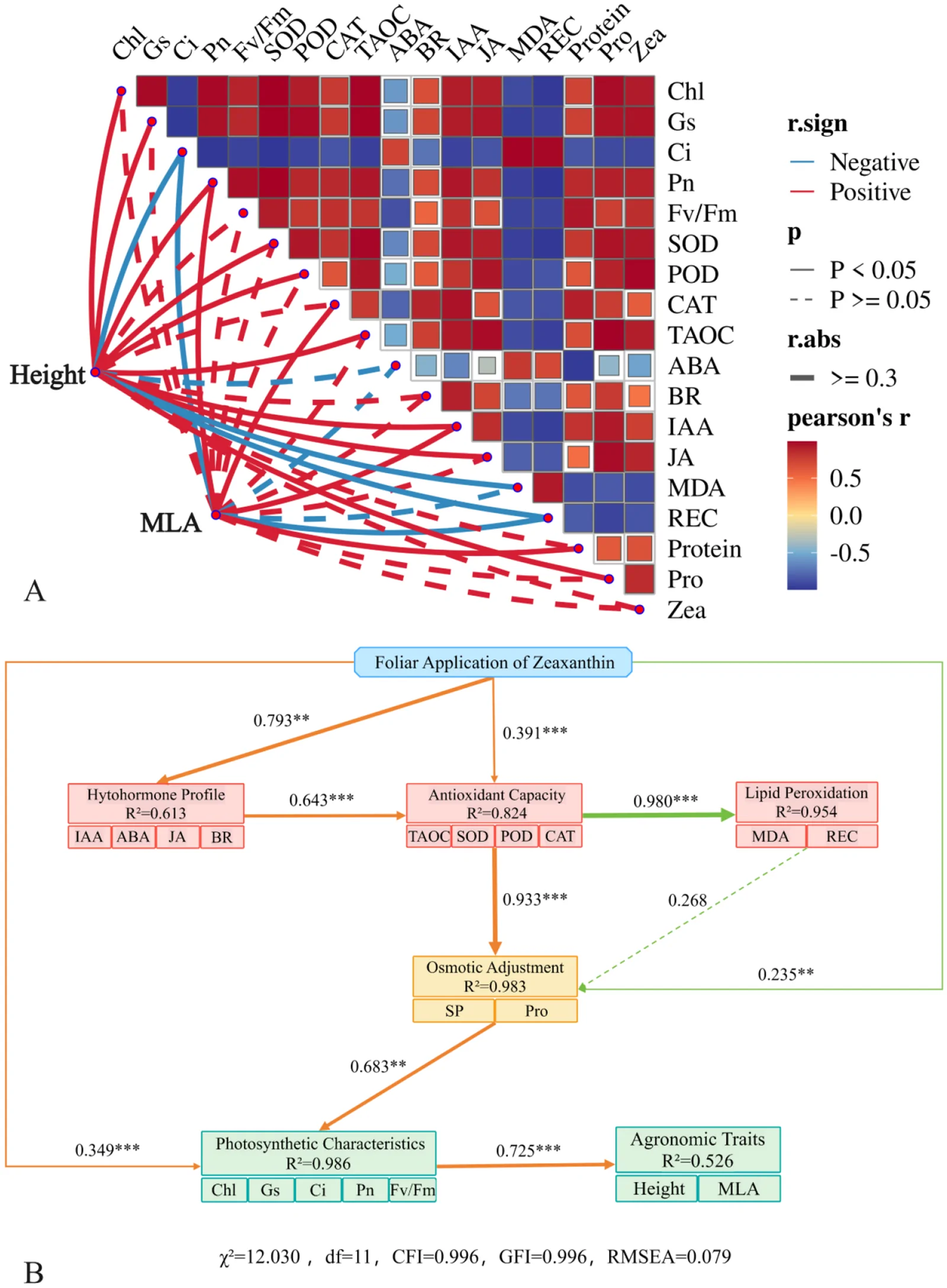
Correlation analysis of physiological traits and structural equation model (SEM)-based pathway analysis of the mechanisms underlying zeaxanthin action. **(A)** Correlation analysis of physiological traits, agronomic traits, and exogenous zeaxanthin concentration. The thickness of the curves indicates the Mantel’s r statistic, whereas the curve colors represent statistical significance. Solid and dashed curves indicate correlations at *P* ≥ 0.05 and *P* < 0.05, respectively. Red and blue curves represent positive and negative correlations, respectively. Right-angled triangles indicate pairwise comparisons among the indicators, with color gradients representing Pearson’s correlation coefficients. **(B)** Structural equation model (SEM) illustrating the regulatory pathways associated with zeaxanthin application. The SEM was constructed based on hormone levels, antioxidant capacity, membrane lipid peroxidation, osmotic adjustment substances, photosynthetic characteristics, and agronomic traits following zeaxanthin pretreatment. Solid and dashed arrows indicate direct and indirect effects, respectively. Orange and green arrows represent positive and negative effects, respectively. The values and widths of the arrows indicate the standardized path coefficients. *, **, and *** indicate significance at *P* < 0.05, *P* < 0.01, and *P* < 0.001, respectively. R² represents the proportion of explained variance. Height, plant height; MLA, maximum leaf area; Chl, chlorophyll content; *Gs*, stomatal conductance; *Ci*, intercellular CO₂ concentration; *Pn*, net photosynthetic rate; *Fv/Fm*, maximum photochemical efficiency of photosystem II; SOD, superoxide dismutase; POD, peroxidase; CAT, catalase; TAOC, total antioxidant capacity; ABA, abscisic acid; BR, brassinosteroid; IAA, indole-3-acetic acid; JA, jasmonic acid; MDA, malondialdehyde; REC, relative electrolyte conductivity; SP, soluble protein; Pro, proline; Zea, exogenous zeaxanthin concentration.

As shown in **Figure 5**, the physiological traits were classified into five functional modules: photosynthetic performance (Chl, *Pn*, *Gs*, *Fv/Fm*, and *Ci*); membrane lipid peroxidation and injury (MDA and REC); antioxidant defense (SOD, POD, CAT, and TAOC); hormonal balance (IAA, ABA, JA, and BR); and osmotic regulation (Protein and Pro). Plant height and maximum leaf area were generally positively correlated with photosynthetic and antioxidant traits but negatively correlated with membrane injury indicators. Zeaxanthin concentration was positively correlated with Chl, *Gs*, *Pn*, *Fv/Fm*, antioxidant traits, JA, BR, IAA, SP, and Pro, whereas it was negatively correlated with *Ci*, ABA, MDA, and REC.

The structural equation model showed a good fit to the data. Zeaxanthin exerted its strongest direct positive effect on hormonal balance, which was positively associated with antioxidant defense. Antioxidant defense was negatively associated with membrane lipid peroxidation and injury but positively associated with osmotic regulation. Zeaxanthin also directly enhanced photosynthetic performance, whereas osmotic regulation further promoted photosynthetic performance. Photosynthetic performance explained 52.6% of the variation in plant height and maximum leaf area. A significant negative direct path from zeaxanthin to osmotic regulation was also detected. Because the SEM represents statistical associations among physiological modules, these paths should not be interpreted as evidence of causal relationships.

### 3.7 Effects of zeaxanthin pretreatment on xanthophyll cycle components in flue-cured tobacco leaves under low-temperature stress and during recovery

**Figure 6:**
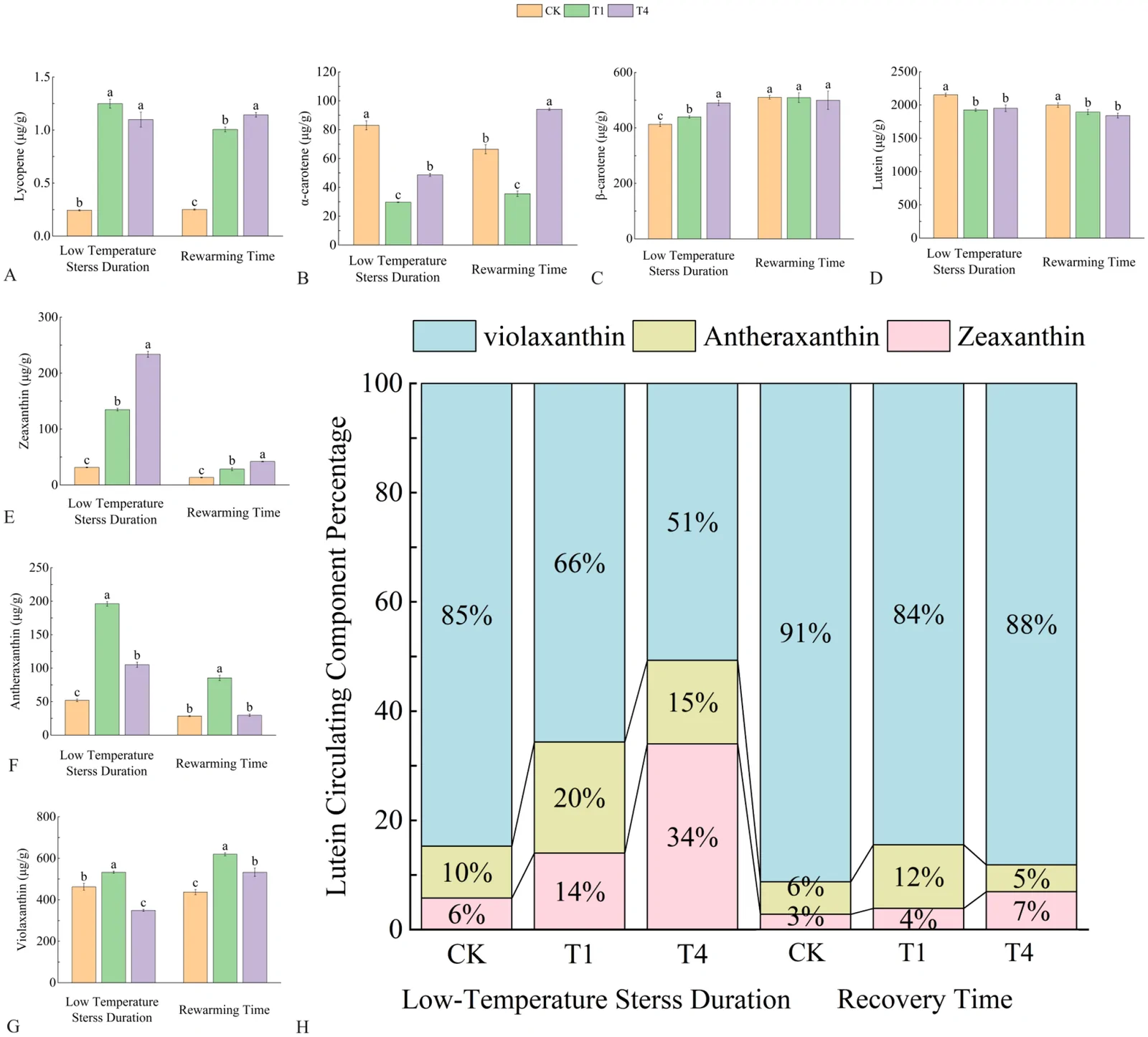
Effects of zeaxanthin pretreatment on xanthophyll cycle components in flue-cured tobacco leaves under low-temperature stress and during recovery. **(A)** Lycopene. **(B)** α-Carotene. **(C)** β-Carotene. **(D)** Lutein. **(E)** Zeaxanthin. **(F)** Antheraxanthin. **(G)** Violaxanthin. **(H)** Relative proportions of zeaxanthin, antheraxanthin, and violaxanthin. Error bars represent the standard error (SE). Different lowercase letters indicate statistically significant differences among treatments at the same growth stage (*p* < 0.05, ANOVA followed by Duncan’s multiple range test).

As shown in **Figure 6**, low-temperature stress significantly altered carotenoid composition. Lycopene content increased in T1 and T4, whereas α-carotene and lutein contents decreased, with the greatest reductions generally observed in T1. β-Carotene content followed the order T4 > T1 > CK.

Low-temperature stress also significantly affected the xanthophyll cycle. Zeaxanthin and antheraxanthin contents increased in T1 and T4 relative to CK, whereas violaxanthin content showed the opposite pattern. Zeaxanthin reached its highest level in T4, increasing by 624.61% relative to CK, whereas antheraxanthin showed its greatest increase in T1. The relative proportions of zeaxanthin and antheraxanthin increased under low-temperature stress, particularly in T4, whereas the relative proportion of violaxanthin decreased.

After rewarming, the contents and relative proportions of zeaxanthin and antheraxanthin declined in T1 and T4 and generally approached CK levels. During recovery, α-Carotene content increased in T4, whereas zeaxanthin content remained relatively high in T4. These results indicate that zeaxanthin pretreatment enhanced xanthophyll-cycle de-epoxidation under low-temperature stress and that this response was partially reversed during recovery.

## 4 Discussion

### 4.1 Zeaxanthin alleviates low-temperature-induced inhibition of photosynthesis and promotes tobacco growth

Low-temperature stress is known to reduce leaf chlorophyll content and photosynthetic capacity, thereby impairing carbon assimilation and restricting plant growth and development (Gan et al., 2019; Gong et al., 2020; Zhang et al., 2021, Zhang et al., 2024). In the present study, exogenous zeaxanthin markedly alleviated the low-temperature-induced inhibition of photosynthesis in flue-cured tobacco. Under low-temperature stress, *Pn* decreased, whereas *Ci* increased suggesting that photosynthesis was affected primarily by non-stomatal limitations. Following zeaxanthin application, Chl, *Gs*, *Pn*, and *Fv/Fm* were significantly increased, whereas *Ci* gradually decreased to levels comparable to those in CK.

This recovery may be associated with the role of zeaxanthin in enhancing photoprotection and maintaining carbon assimilation under stress conditions. Under low-temperature conditions, excess excitation energy can damage the PSII reaction center, as reflected by the marked decline in *Fv/Fm* in T1. Zeaxanthin may help stabilize thylakoid membranes and support *NPQ*-mediated thermal dissipation (Gao et al., 2010; Demmig-Adams et al., 2020). Consistent with this interpretation, *NPQ* was significantly higher in the zeaxanthin-pretreated treatments than in T1 under low-temperature stress, with the greatest increase observed in T4 (**Table 1**). Enhanced *NPQ* may dissipate excess excitation energy, thereby reducing photooxidative damage and chlorophyll degradation (Xi et al., 2025).

Meanwhile, *Gs* increased and *Ci* decreased following zeaxanthin treatment. This pattern may indicate that the availability of CO_2_ for the Calvin cycle improved after the recovery of the light reactions, thereby enhancing carbon assimilation efficiency (von Caemmerer et al., 2025). Overall, these findings suggest that exogenous zeaxanthin may improve the ability of tobacco seedlings to cope with low-temperature stress by promoting the recovery of photosynthetic function and carbon assimilation, which may subsequently support plant growth (Zhang et al., 2025).

### 4.2 Zeaxanthin may alleviate membrane lipid peroxidation by enhancing antioxidant defense and osmotic regulation

Low-temperature stress can disturb cellular redox homeostasis and lead to the accumulation of ROS and membrane lipid peroxidation, thereby compromising membrane integrity (Bonnecarrère et al., 2011). In the present study, zeaxanthin treatment increased the activities of SOD, POD, and CAT, as well as TAOC levels. These results suggest that the protective effect of zeaxanthin may be associated with enhanced antioxidant defense (Nna et al., 2019; Tang et al., 2021).

This effect may be partly attributable to the structural properties of zeaxanthin. Its conjugated double-bond system has been reported to confer strong ROS-scavenging capacity, including the ability to quench singlet oxygen (^1^O_2_) (Shanaida et al., 2025). Zeaxanthin may also participate in the regulation of antioxidant enzyme activity, thereby limiting lipid peroxidation. Consistent with this interpretation, the reductions in MDA content and REC observed in the present study suggest that membrane oxidative damage and disruption of membrane permeability were alleviated to some extent (Pamplona, 2011).

In addition, zeaxanthin treatment was associated with increased levels of soluble protein and free proline, which may reflect an enhanced capacity for osmotic regulation under low-temperature stress (Liu et al., 2019). These osmolytes are generally considered important contributors to cellular homeostasis because they help maintain turgor pressure and stabilize cellular membranes. Collectively, these findings suggest that zeaxanthin may mitigate low-temperature-induced membrane damage, at least in part, by strengthening antioxidant defense and osmotic regulation. Nevertheless, the molecular mechanisms underlying these effects require further investigation.

### 4.3 Zeaxanthin-induced alleviation of low-temperature stress is associated with adjustments of the hormonal network

Plant adaptation to low-temperature stress is governed by coordinated hormonal crosstalk and redox regulation rather than by a single signaling pathway. In the present study, exogenous zeaxanthin was associated with increased levels of JA, IAA, and BR and with reduced ABA levels under low-temperature stress. This hormonal reprogramming suggests that zeaxanthin may participate in the regulation of defense- and growth-related signals during low-temperature stress.

The increase in JA may indicate enhanced JA-mediated defense signaling. JA is known to promote antioxidant defense and the accumulation of secondary metabolites, thereby alleviating reactive oxygen species overproduction and membrane lipid peroxidation under stress conditions (Zhou and Memelink, 2016; Wasternack and Song, 2017). The concomitant increase in IAA suggests that zeaxanthin may also influence the balance between growth and defense under low-temperature stress. Because JA signaling is often associated with the suppression of auxin-mediated growth, this hormonal adjustment may help redirect resources toward protective responses (Huot et al., 2014; Shi et al., 2014). The elevated BR level further supports this interpretation. BRs have been reported to enhance low-temperature tolerance by improving antioxidant capacity, stabilizing photosystems, and inducing stress-responsive gene expression through *BZR1/BES1*-dependent pathways (Tang et al., 2011; Anwar et al., 2018).

In contrast, ABA levels declined after zeaxanthin treatment. ABA is a well-established positive regulator of cold acclimation, and this reduction may initially appear counterintuitive. However, three considerations may help explain this observation. First, zeaxanthin and ABA are derived from a common xanthophyll precursor pool. Violaxanthin is de-epoxidized to form zeaxanthin through the xanthophyll cycle and also serves as a substrate for 9-cis-epoxycarotenoid dioxygenase (NCED) during ABA biosynthesis (North et al., 2007; Cardoso et al., 2020; Perreau et al., 2020). The enhanced de-epoxidation toward zeaxanthin observed in this study may therefore have reduced the availability of precursors for ABA production. Second, ABA accumulation is typically triggered by the severity of stress. Zeaxanthin-pretreated plants exhibited less membrane damage, as indicated by their lower MDA content and REC. Therefore, the weaker ABA response may reflect reduced stress perception rather than impaired defense. Third, sustained high ABA levels promote stomatal closure, thereby restricting CO_2_ uptake and carbon assimilation (Chaves et al., 2009; Agurla et al., 2018). A moderate reduction in ABA may therefore have helped maintain stomatal conductance and photosynthetic carbon gain under chilling conditions, consistent with the higher *Gs* and *Pn* and lower *Ci* observed in the T3 and T4. It should also be noted that ABA content does not necessarily reflect ABA signaling capacity; downstream ABA signaling may have remained functional in zeaxanthin-pretreated plants (Sun et al., 2022).

The relationship between reduced ABA content and improved stomatal conductance in this study was correlative. The concurrent recovery of *Gs* and *Pn* in zeaxanthin-pretreated plants was consistent with this interpretation. In addition, ABA content was significantly negatively correlated with *Gs* at most sampling times. These results provide correlative support for the role of reduced ABA levels in maintaining stomatal opening. However, direct evidence of causality is still lacking. In particular, ABA-Gs dose-response relationships, NCED expression, and ABA metabolite profiles were not assessed. These limitations should therefore be acknowledged, as discussed in Section 4.5.

### 4.4 Zeaxanthin-induced enhancement of low-temperature tolerance is associated with adjustments of the xanthophyll cycle

The xanthophyll cycle enables plants to dissipate excess excitation energy as heat through non-photochemical quenching (*NPQ*), thereby reducing ROS accumulation and helping maintain photosystem function under photooxidative stress. In the present study, low-temperature stress appeared to activate this pathway. Zeaxanthin and antheraxanthin levels increased, suggesting that tobacco seedlings initiated a basic photoprotective response to alleviate cold-induced photoinhibition (Gao et al., 2010; Verhoeven, 2014; Sun et al., 2018).

Exogenous zeaxanthin further enhanced this response, consistent with changes in carotenoid partitioning within the xanthophyll cycle. Specifically, zeaxanthin treatment increased leaf zeaxanthin content and reduced violaxanthin content, whereas antheraxanthin content also increased under stress, particularly in the treatment showing the strongest response. This pattern suggests that exogenous zeaxanthin promoted xanthophyll-cycle de-epoxidation, favoring the conversion of violaxanthin toward zeaxanthin and reducing the upstream violaxanthin pool (Short et al., 2024).

This metabolic shift may support cold acclimation through at least two mechanisms. First, the enlarged zeaxanthin pool may strengthen *NPQ*-mediated thermal dissipation in PSII. This interpretation is consistent with the recovery of *Fv/Fm* observed in Section 3.2 and with the established role of xanthophyll cycle-dependent photoprotection in tobacco (Gao et al., 2010; Kromdijk et al., 2016; Głowacka et al., 2018). It is also supported by the higher *NPQ* values measured in the zeaxanthin-pretreated treatments than in T1 (**Table 1**). Second, violaxanthin is a direct precursor of ABA biosynthesis (Cardoso et al., 2020; Perreau et al., 2020); therefore, its lower abundance may partly explain the reduced ABA levels observed in Section 3.5. This finding may indicate a substrate-level trade-off in carotenoid metabolism, in which enhanced xanthophyll cycling prioritizes photoprotection over ABA production (Liu et al., 2023). A moderate reduction in ABA may not necessarily weaken stress adaptation. Instead, it may help prevent excessive ABA-mediated stomatal closure and maintain CO_2_ availability and carbon assimilation under cold conditions (Agurla et al., 2018; Fatma et al., 2021).

**Figure 7:**
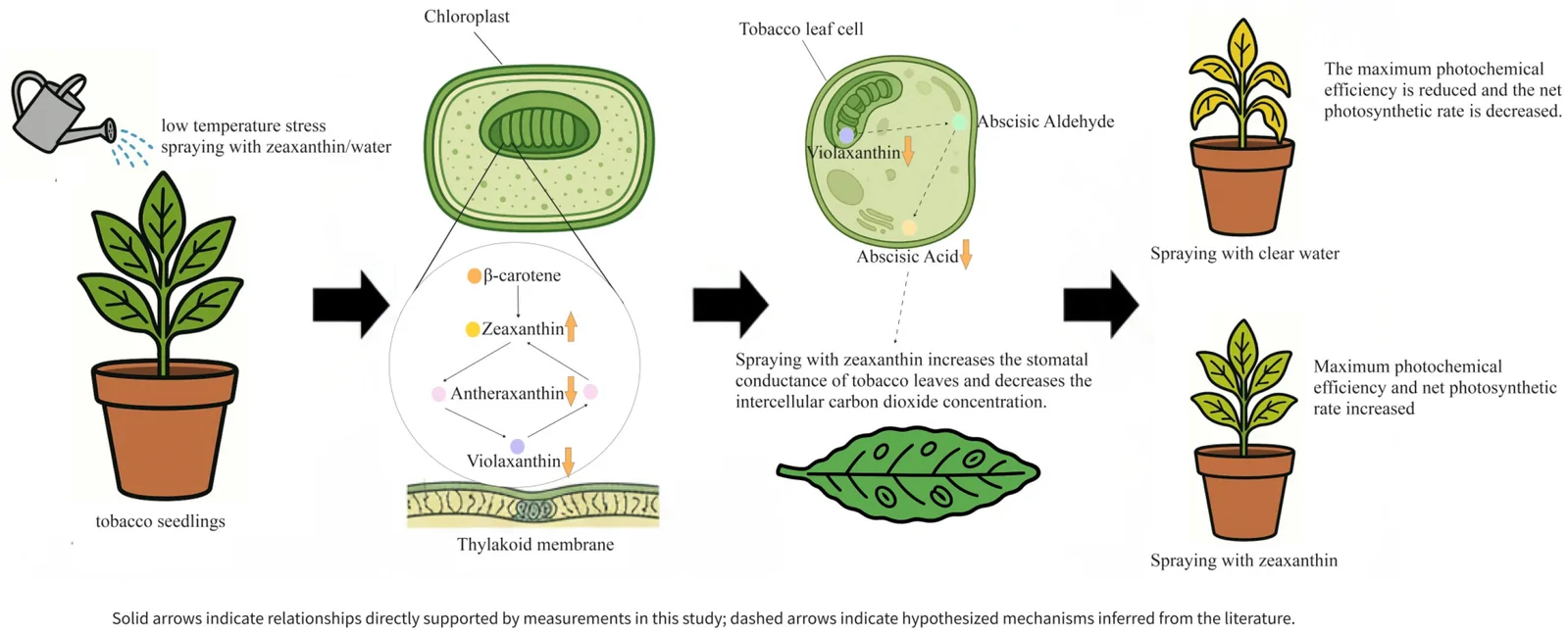
Hypothetical model by which zeaxanthin pretreatment alleviates low-temperature stress in flue-cured tobacco seedlings through carotenoid metabolism and growth-related traits. Upward arrows indicate increased content, and downward arrows indicate decreased content. Solid arrows indicate relationships directly supported by measurements in this study (increased leaf zeaxanthin content and de-epoxidation state; enhanced *NPQ*; recovery of Fv/Fm and photosynthetic gas exchange; enhanced antioxidant enzyme activities; reduced MDA content, REC, and ABA content), whereas dashed arrows indicate hypothesized links inferred from previous literature (*NPQ*-mediated thermal dissipation protecting PSII; substrate competition between the xanthophyll cycle and ABA biosynthesis; ABA-mediated stomatal regulation).

This interpretation is based on steady-state metabolite measurements and does not directly resolve metabolic fluxes or the regulatory hierarchy. Future studies should combine transcript profiling, enzyme activity assays, and isotope tracing to clarify how exogenous zeaxanthin affects xanthophyll-cycle remodeling, ABA biosynthesis, and cold adaptation at the mechanistic level. Such analyses will be necessary to establish a causal framework linking carotenoid partitioning to improved cold tolerance in flue-cured tobacco. Based on the physiological, biochemical, hormonal, and carotenoid-related responses observed in this study, we propose a hypothetical model illustrating how zeaxanthin pretreatment may alleviate low-temperature stress through enhanced xanthophyll-cycle de-epoxidation, NPQ-mediated photoprotection, antioxidant defense, osmotic regulation, and hormonal adjustment (**Figure 7**).

From a practical perspective, foliar application of zeaxanthin at the nursery stage is technically simple and compatible with existing flue-cured tobacco production. Chilling episodes during transplanting can often be forecast several days in advance, allowing preventive spraying. In this study, an application of 75 mg L^-1^ at 2 mL per plant was effective, corresponding to a small amount of active ingredient per plant. Nevertheless, the relatively high cost of purified zeaxanthin may limit its large-scale field application. Zeaxanthin-rich natural extracts, such as wolfberry or goji berry extracts, and formulated biostimulant combinations, such as zeaxanthin combined with brassinolide, may provide more cost-effective alternatives (Ding et al., 2022; Li et al., 2022). Field validation under natural chilling conditions, together with a cost-benefit analysis, is required before practical recommendations can be made.

### 4.5 Limitations and future perspectives

First, the experimental design did not include a zeaxanthin-only treatment under normal-temperature conditions. Comparisons between T1 and the zeaxanthin treatments under identical chilling conditions support the conclusion that zeaxanthin alleviates chilling injury. However, the possibility that zeaxanthin also promotes growth under non-stress conditions cannot be excluded. Because zeaxanthin is an endogenous photosynthetic pigment whose photoprotective function depends on light and stress conditions, its effects under optimal temperatures may be limited. Nevertheless, future studies should include a zeaxanthin-only control under normal-temperature conditions to distinguish chilling-specific effects from general physiological effects.

Second, the mechanistic interpretations in this study are based largely on correlations among physiological modules. Although structural equation modeling revealed a coherent covariance structure that was consistent with the proposed pathway, it does not establish causality. Therefore, the path coefficients should be regarded as hypotheses requiring future validation through genetic or pharmacological approaches, including the use of VDE or ZEP inhibitors and mutants.

Third, the mechanism by which foliar-applied zeaxanthin enters leaves and reaches chloroplasts remains unverified. Although foliar zeaxanthin application has been shown to increase leaf zeaxanthin content in pepper (Tang et al., 2021; Ding et al., 2022), its subcellular fate was not resolved in those studies. Accordingly, part of the increase in leaf zeaxanthin content observed here may reflect direct deposition and retention of the applied pigment in leaf tissues rather than *de novo* synthesis or enhanced xanthophyll cycling. Follow-up studies using isotope-labeled zeaxanthin or epidermis- and mesophyll-fractionation techniques would help clarify its uptake, transport, and subcellular distribution.

Fourth, endogenous hormone levels were measured using commercial ELISA kits, which provide relative rather than absolute quantification. Confirmation of the key changes in hormone levels, particularly those of ABA and JA, using LC-MS/MS would further strengthen the conclusions regarding hormonal regulation.

## 5 Conclusion

Exogenous zeaxanthin effectively alleviated low-temperature-induced injury in flue-cured tobacco seedlings. Among the tested treatments, T4 (75 mg L^−1^) produced the most pronounced effects. It significantly improved seedling growth and agronomic performance during both the chilling and recovery stages. In addition, zeaxanthin enhanced photosynthetic capacity and non-photochemical quenching, increased antioxidant enzyme activities, reduced membrane lipid peroxidation, and helped maintain a more favorable endogenous hormone balance.

Structural equation modeling further indicated that the protective effects of zeaxanthin were associated with direct and indirect relationships among physiological modules. Zeaxanthin application was positively associated with hormonal regulation, antioxidant capacity, and photosynthetic performance. Enhanced photoprotection was also associated with changes in xanthophyll-cycle components, which may be related to the regulation of abscisic acid.

Overall, zeaxanthin represents a potentially effective strategy for improving cold tolerance in tobacco seedlings. Its protective effects may involve the coordinated regulation of physiological and biochemical responses to low-temperature stress and the promotion of post-stress recovery.

## Data Availability

The original contributions presented in the study are included in the article/supplementary material. Further inquiries can be directed to the corresponding authors.
